# Harlequin syndrome during peripheral cardiopulmonary bypass in a patient with an obstructing tracheal schwannoma: A case report

**DOI:** 10.1002/ccr3.7509

**Published:** 2023-06-13

**Authors:** Cynthia Karam, Nancy Abou Nafeh, Marie T. Aouad, Sahar Siddik‐Sayyid, Roland Kaddoum, Carine Zeeni, Sandra Anka, Bashir Shaya, Amro Khalili

**Affiliations:** ^1^ Department of Anesthesiology and Pain Medicine American University of Beirut Medical Center Beirut Lebanon

**Keywords:** differential hypoxemia, femorofemoral cardiopulmonary bypass (CPB), harlequin syndrome, north–south syndrome, venoarterial (VA) extracorporeal membrane oxygenation (ECMO), venovenous (VV) extracorporeal membrane oxygenation (ECMO)

## Abstract

Surgical resection of obstructive tracheal tumors can be challenging to cardiothoracic surgeons and anesthesiologists. It is often difficult in these cases to maintain oxygenation by face mask ventilation during induction of general anesthesia. Also, the extent and location of these tracheal tumors can preclude conventional induction of general anesthesia and subsequent successful endotracheal intubation. Peripheral cardiopulmonary bypass (CPB) under local anesthesia and mild intravenous sedation may be safe to support the patient until securing a definitive airway. We describe a case of a 19‐year‐old female with a tracheal schwannoma, who developed differential hypoxemia (Harlequin, or North–South, syndrome) after institution of awake peripheral femorofemoral venoarterial (VA) partial CBP.

## Graphical abstract

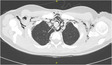

## 1 INTRODUCTION

Tracheal tumors become symptomatic in most patients when they occupy more than 80% of the tracheal lumen. Many modalities, such as high‐frequency jet ventilation in spontaneously breathing patients, low‐lying tracheostomies, and laser resection have been described for ventilation and definitive airway establishment in patients undergoing surgical resection of these tumors.^1^, ^2^ It is a challenge to anesthetize and even surgically resect these tumors when they occupy most of the tracheal lumen, especially when they are located lust above the carina, even without bronchial involvement.^3^ Peripheral cardiopulmonary bypass (CPB) was instituted and described as a safe method to provide adequate oxygenation support during such surgeries.^3^, ^4^, ^5^, ^6^, ^7^ In our case, a tracheal schwannoma was resected with the institution of peripheral femorofemoral venoarterial (VA) partial CPB in an awake patient under local anesthesia and mild intravenous sedation. Harlequin, or North–South, syndrome (i.e., desaturation of the upper half of the body with well‐saturated blood supplying the lower half) with its associated risks of myocardial and cerebral ischemia, along with other challenges were encountered and troubleshooted through multidisciplinary coordination between different teams.

## 2 CASE PRESENTATION

Our patient was a 19‐year‐old female who weighed 55 kg and complained of progressively worsening dyspnea, dry cough, hoarseness, and noisy breathing over the past few hours prior to presentation to the emergency department in our institution. She was a non‐smoker with a negative medical and surgical history and no food or drug allergies. She had been experiencing similar but milder symptoms over the past 6 months and was treated as an asthmatic patient.

On physical examination, she was found to be grossly dyspneic with an audible inspiratory stridor, able to breathe only in the semi‐sitting (45° head‐up tilt) position, with clear lungs on auscultation. She was hemodynamically stable with a pulse oximetric oxygen saturation (SpO_2_) value of 97% on 6 L/min supplemental oxygen by face mask. Ear, nose, and throat (ENT) surgical team performed flexible fiberoptic laryngoscopy through the left nasal cavity that was anatomically unremarkable down to the vocal cords. However, the scope could not be negotiated distal to the vocal cords.

**FIGURE 1:**
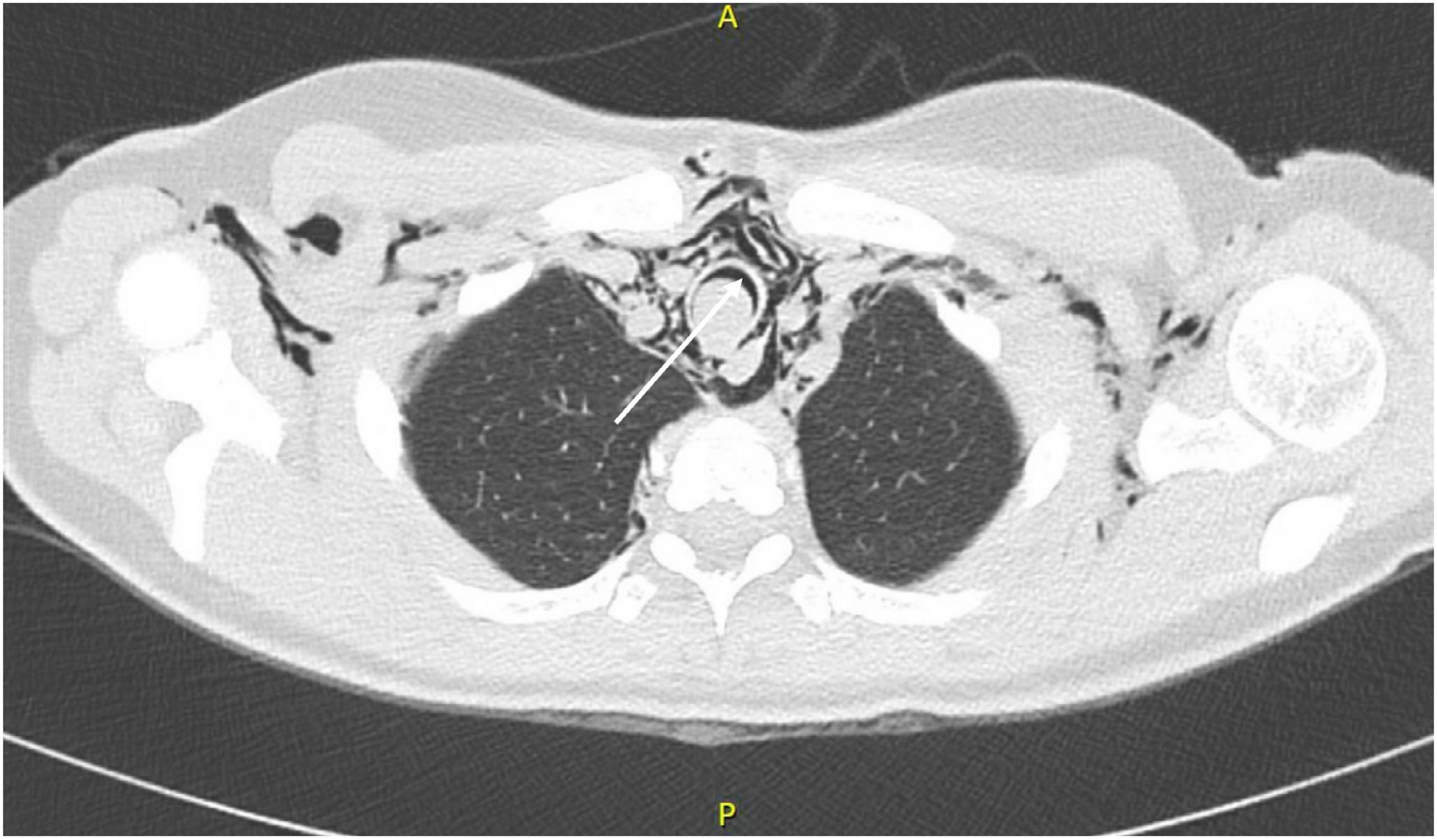
Chest computed tomography (CT) scan. White arrow shows near total obstruction of the tracheal lumen by the tracheal schwannoma.

Further evaluation with chest X‐ray revealed subcutaneous emphysema at the neck and anterior chest wall along with moderate sized pneumomediastinum. Chest computed tomography (CT) scan showed a 1.7 × 1.4 cm well circumscribed, lobulated, homogenously enhancing intratracheal soft tissue lesion arising from the posterior wall of the trachea, projecting into the tracheal lumen and causing 90% luminal narrowing, 5.3 cm above the carina, and at T1 level (Figure 1).

After discussion with the ENT and cardiothoracic surgical teams, the decision was made to immediately proceed with diagnostic and therapeutic rigid bronchoscopy for tumor biopsy and bipolar electrocautery‐assisted resection with the support of peripheral femorofemoral VA partial CPB under local anesthesia and mild intravenous sedation.

The ENT surgeon was wise not to proceed without peripheral CPB support because of severe narrowing of the trachea, unknown nature of the tumor, and risk of tumor bleeding, rupture, or complete downstream airway obstruction if conventional general anesthesia and endotracheal intubation was attempted. The patient and her family were rapidly counseled and consented to the proposed surgical plan.

Upon presentation to the operating room, the patient was very anxious, agitated, and only tolerating the semi‐sitting position while on 12 L/min supplemental oxygen by non‐rebreather face mask with a SpO_2_ value of 98% measured using a pulse oximetry probe attached to her left hand. Three 20‐G peripheral intravenous lines and a 20‐G right radial arterial catheter were secured.

While in the semi‐sitting position and with a wedge placed under her groins, the cardiothoracic surgeon attempted cannulation of the common femoral vessels under ultrasound guidance with generous local anesthetic infiltration (200 mg of 1% lidocaine) to each groin and carefully titrated intravenous sedation (1 mg of midazolam and 50 mcg of fentanyl in total). The right common femoral vein was cannulated percutaneously with a 21‐Fr multi‐stage venous cannula. The position of its tip in the right atrium (RA) was estimated by measuring the distance from the groin to the mid right sternal border. The left common femoral artery could not be cannulated percutaneously with a 17‐Fr arterial cannula because of its relatively small size and the unfavorable semi‐setting position. This was complicated by an arterial hematoma and thus was aborted. The right common femoral artery was surgically exposed via a groin incision and directly cannulated, this time with a smaller 15‐Fr arterial cannula.

**FIGURE 2:**
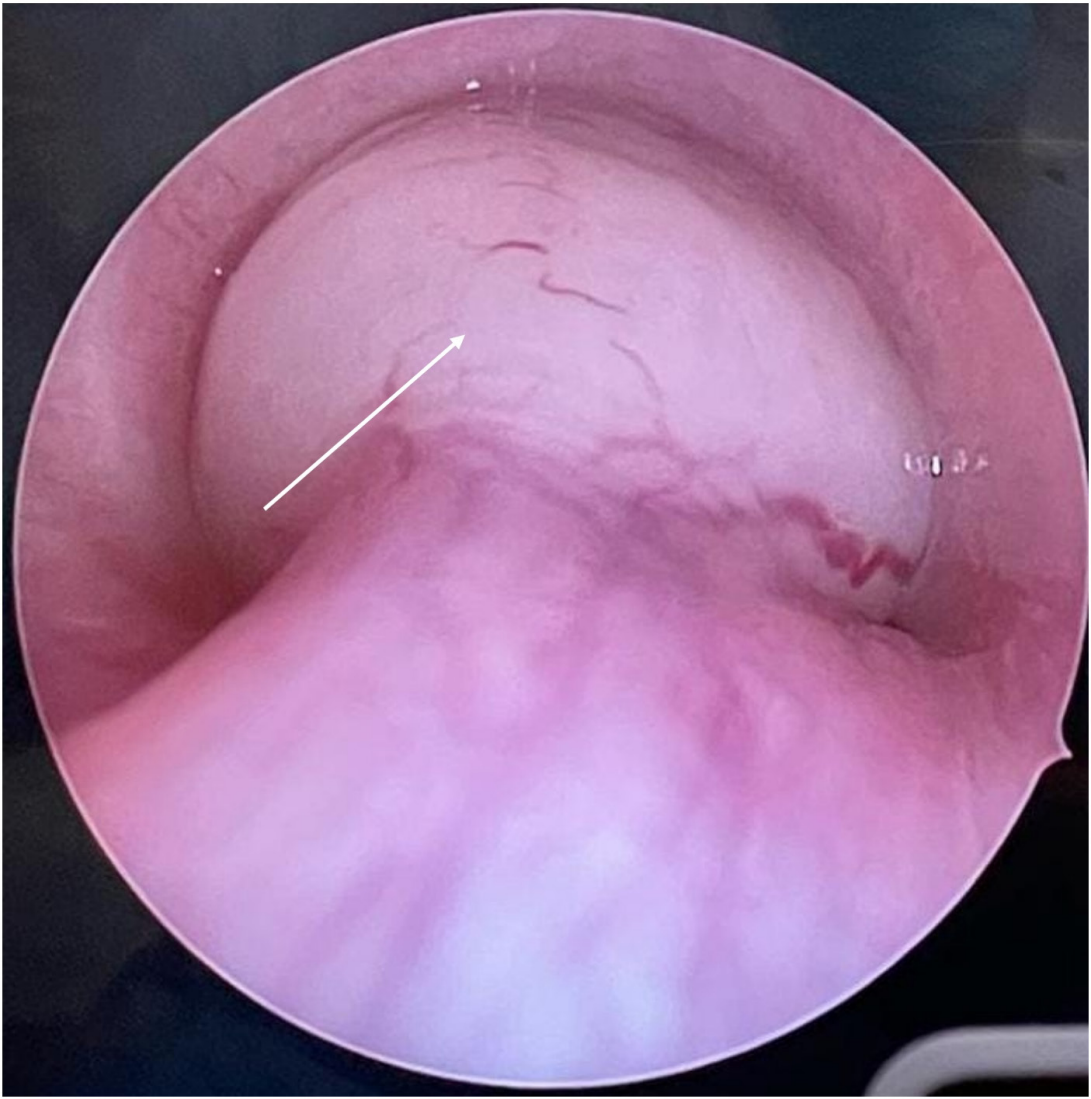
Intraoperative rigid bronchoscopy image of the tracheal schwannoma (white arrow).

This was followed by the institution of normothermic partial (75%) flow femorofemoral VA CPB after achieving an activated clotting time (ACT) of 480 s by systemic heparinization. Afterwards, 50 mg lidocaine, 2 mg midazolam, 150 mcg fentanyl, 100 mg propofol, 8 mg dexamethasone, and 50 mg rocuronium were administered intravenously for full general anesthesia so that the ENT surgeon can subsequently do rigid bronchoscopy for tumor biopsy and resection in the supine position (Figure 2).

Five minutes after initiation of CPB, the first sample of arterial blood taken from the femoral arterial perfusion line of the CPB machine showed a pH of 7.35, partial pressure of oxygen (PaO_2_) of 469 mmHg while on 70% fraction inspired of oxygen (FiO_2_), arterial oxygen saturation (SaO_2_) of 99%, and partial pressure of carbon dioxide (PaCO_2_) of 34.9 mmHg. However, 20 min later, and while the ENT surgeon was downsizing the tracheal tumor, we noticed generalized ST segment depression that was most evident on lead II of the electrocardiogram. Immediate inspection of the right femoral arterial perfusion line by the perfusionists showed adequate, bright oxygenated blood indicating normally functioning parts of the CPB machine. Also, the values of her mixed venous oxygen saturation (SvO_2_) in the femoral venous return line of the CPB machine and her SpO_2_ on the left hand were stable all the time at 75% and 99%, respectively. So, the decision was to withdraw a new arterial blood sample, this time from her right radial arterial catheter. It showed a pH of 7.25, PaO_2_ of 20 mmHg while on 70% FiO_2_, SaO_2_ of 25%, and PaCO_2_ of 56 mmHg. The diagnosis of differential hypoxemia (Harlequin, or North–South, syndrome) was made and the perfusionists attempted to achieve full (100%) flow for a brief period to achieve adequate gas exchange, but they could not because of her normally functioning heart that was beating, as no cardioplegia was given in her case.

After rapid communication between all teams, and as the tracheal tumor was greatly downsized by that time to 50% of its original size, we attempted endotracheal intubation, using a 7 mm internal diameter endotracheal tube (ETT), with subsequent successful lung ventilation. Shortly after endotracheal intubation, repeated right radial arterial blood gas analysis showed a pH of 7.36, PaO_2_ of 310 mmHg while on 100% FiO_2_, SaO_2_ of 99.8%, and PaCO_2_ of 39 mmHg, suggesting that Harlequin syndrome was corrected by adequate pulmonary gas exchange and oxygenation of the venous blood entering her heart.

The ENT surgeon continued tumor debulking. However, complete tumor resection was not possible bronchoscopically because of the fear of tumor fragmentation and loss of debris in the distal airway.

At the end of the procedure, heparin was reversed with protamine and the patient was smoothly weaned off CPB after confirming normal ventilation. Her femoral vessels were decannulated and she was then transferred to the intensive care unit (ICU) with stable vital signs, sedated, intubated, and mechanically ventilated. CPB duration was 110 min, and the surgery lasted 85 min. Few hours later, the patient regained full level of consciousness with a totally normal neurological examination. The ENT and ICU teams took the decision to keep her intubated until a clear management plan is devised.

Histopathology examination was suggestive of a benign tracheal schwannoma. Few days later, the tracheal segment incorporating the schwannoma was totally resected en block followed by primary end‐to‐end anastomosis and reconstruction of the trachea. Here, successful cross‐field ventilation was done through inserting a sterile ETT by the ENT surgeon distal to the residual tracheal schwannoma and proximal to the tracheal carina. The patient was then extubated in the operating room in a stable condition and transferred to the ICU for close monitoring for 3 days before safely discharging her home.

## 3 DISCUSSION

Primary tracheal tumors are rare and comprise only 1% of all neoplasms. Among these only 25% are benign.^8^ Schwannoma is an uncommon benign tracheal tumor, and thus tracheal schwannomas are extremely rare.^8^ Since schwannomas are slow growing tumors of the trachea, they produce symptoms only when they are large enough to cause obstructive symptoms. Since they present with dyspnea and dry cough, they are often mistreated for years as asthma^9^ or chronic obstructive pulmonary disease, until an endotracheal lesion is suspected due to unresponsiveness to inhaled medications or by the appearance of the flow volume loop or when the symptoms progress to stridor, like it did in our patient.

When near total obstruction of the tracheal lumen occurs, attempts at inserting an endotracheal tube or flexible fiberoptic bronchoscope may cause complete occlusion of the airway and respiratory arrest. Subsequent recovery from this acute deterioration may be very difficult with catastrophic outcomes.

The use of extracorporeal circuits to support pulmonary function during resection of a primary tracheal tumor was initially reported in 1961 but other reports with successful outcomes followed.^4^, ^5^, ^6^, ^7^, ^10^, ^11^, ^12^, ^13^ This technique provides a method of maintaining normal gas exchange allowing time for resection and good surgical access even if complete tracheal obstruction occurs.^11^, ^14^ CPB is well described as an adjunct to tracheal tumor resection but is rarely instituted under local anesthesia and mild intravenous sedation.^15^, ^16^ Two cases of thyroid lymphoma causing near complete airway obstruction have been reported where the use of a portable CPB circuit was used in one case and conventional femorofemoral VA CPB under local anesthesia in the other to assist airway management and provide a safe resection.^17^, ^18^ Chiu et al. and Misra et al. reported the use of temporary CPB using femoral artery and vein cannulation under spinal anesthesia for tracheal resection and reconstruction.^7^, ^19^ Also, Kar et al. reported the use of temporary CPB using right femoral artery and vein cannulation, for tracheal tumor resection, after a right femoral nerve block was administered.^5^

Despite the fact that CPB ensures adequate gas exchange, concerns have been raised regarding CPB‐related complications, such as lower limbs ischemia, swelling, and the propensity to develop deep vein thrombosis. Major complications include intrapulmonary hemorrhage, coagulopathy, and neurological deficits especially if surgical dissection and bypass times are extensive.^19^, ^20^, ^21^

Our patient presented with progressively worsening dyspnea and audible stridor due to an intraluminal tracheal schwannoma causing near total obstruction. The use of cross‐field ventilation, ventilation through a low‐lying tracheostomy, or endobronchial ventilation was ruled out by the ENT surgeon, due to the site of the tumor near the carina and near total degree of tracheal lumen obstruction. Alternate methods of oxygenation like jet ventilation were also not considered because this may cause dislodgement of a part of the tumor or may change the position of the tumor leading to further luminal obstruction. Moreover, high‐frequency jet ventilation may cause inadequate time for expiration leading to further air trapping, pneumothorax, and worsening pneumomediastinum.^5^ Laser resection was not opted as any bleeding or edema during tumor manipulation can be catastrophic.

Therefore, it was decided to institute femorofemoral bypass in the semi‐sitting position because of absolute inability of the patient to lie flat. Femorofemoral bypass in the semi‐sitting position can have several problems like kinking of the venous cannula and inadequate venous return.^5^ In our case, even after making the patient's position supine, we could not achieve full flow because of the preserved native cardiac output of the patient rather than a problem in the venous cannula resulting in inadequate venous return.

CPB provides the ENT surgeon sufficient time for adequate tumor resection. Most cases described in literature have a short bypass time. Establishing early ventilation once under CPB leads to early weaning from bypass.^5^

Currently, there is no clear consensus on the management of critical subglottic airway obstruction. In these cases, CPB and extracorporeal membrane oxygenation (ECMO) are reasonable methods to use.^4^ Two types of extracorporeal support are present, which are VA and venovenous (VV). VA extracorporeal support is described as the extracorporeal oxygenator is being parallel to the patient's lungs and involves drainage of venous blood followed by oxygenation and return to a peripheral or central artery.^4^ VV extracorporeal support is described as the oxygenator is being in series with the patient's lungs and involves taking venous blood either from the superior vena cava (SVC) or the inferior vena cava (IVC) followed by oxygenation and return to the other vena cava.^4^ VV extracorporeal support in the form of VV ECMO can be used in cases where there is adequate function of the heart and a need for ventilatory support only (e.g., in cases of respiratory failure).^4^ On the other hand, VA ECMO support can be used in cases where there is inadequate function of both the heart and lungs (e.g., in cases of cardiogenic shock).^4^, ^22^

In our patient, VV ECMO support was initially considered using the femoral vein‐right internal jugular vein (IJV) approach. However, the cardiothoracic surgeon estimated that our adolescent patient was very anxious, and it was believed that she would not cooperate with the insertion of a 19‐ or 21‐Fr venous cannula in her neck using local anesthetic infiltration and mild intravenous sedation. Furthermore, the greatly increased cost of the VV ECMO circuit as compared to the traditional VA CPB circuit was another obstacle for its use in her case.

Our patient developed differential hypoxemia (Harlequin, or North–South, syndrome) while on femorofemoral VA CPB support after general anesthesia was induced. This is rare, as most cases reported in the literature describe Harlequin syndrome while on femorofemoral VA ECMO support, not on femorofemoral VA CPB support.^7^ An important difference between the two techniques is the venous reservoir, which is present in VA CPB support. It allows for complete heart emptying, ensuring that only the CPB circuit pumps all the cardiac output.^7^

**FIGURE 3:**
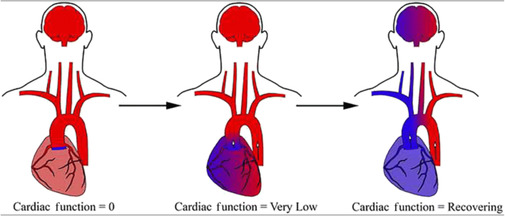
The watershed zone site in Harlequin, or North–South, syndrome is determined by the native LV function, with better native LV function resulting in a watershed zone being more distal. (Prisco AR, et al. Concomitant Respiratory Failure Can Impair Myocardial Oxygenation in Patients with Acute Cardiogenic Shock Supported by VA‐ECMO. J Cardiovasc Transl Res. 2022; 15 (2):217–226. doi:10.1007/s12265‐021‐10110‐2. Springer Nature. http://creativecommons.org/licenses/by/4.0/. No changes were made to the figure).

Harlequin syndrome develops when native left ventricular (LV) function is preserved or is recovering while the lungs' function is still poor (e.g., in respiratory failure) or mechanical ventilation is temporarily paused.^22^, ^23^, ^24^, ^25^ If peripheral femorofemoral VA ECMO is instituted in cardiogenic shock patients, oxygenated blood will be delivered mainly retrograde up to the aortic root by the arterial cannula in the common femoral artery. With native LV function improving, deoxygenated blood exiting the LV because of inadequate pulmonary gas exchange will be pumped back into the arterial circulation. This will create a watershed zone, with an upper body getting deoxygenated blood exiting the LV and a lower body getting oxygenated blood from the arterial cannula.^22^, ^23^, ^24^ The watershed zone site will be determined by the native LV function, with better native LV function resulting a watershed zone being more distal^26^ (Figure 3). In fact, Harlequin syndrome is a sign of recovery of native LV function while on femorofemoral VA ECMO support.^22^

Depending on the magnitude of native LV output, the location of the meeting zone or interface between retrograde flow and antegrade flow will vary, thus oxygenated blood exiting the femoral arterial cannula may not reach the aortic root and aortic arch. This results in coronaries and carotid arteries being perfused with deoxygenated blood, which leads to heart and brain hypoxia.^7^ This is characterized by normal oxygen saturations in the lower body and low oxygen saturations in the upper body. A right radial artery catheter can reliably determine the location of the watershed zone being distal or proximal to the brachiocephalic artery for adequacy of brain oxygenation by serving as a sampling site for arterial blood gas values. Furthermore, near infrared spectroscopy (NIRS) may be used to monitor brain oxygenation.^4^

Based on SpO_2_ values, ST segment depressions, and arterial blood gas findings in our patient we concluded that Harlequin syndrome is occurring with a watershed zone located in the aortic arch, somewhere between the innominate artery and the left subclavian artery. Despite the desaturation, a healthy circle of Willis in our young patient could have contributed to adequate bilateral cerebral oxygenation through her left common carotid artery. Although previous case reports described attempts of augmenting pump flows to overcome the desaturation,^5^ achieving full (100%) flows with peripheral femorofemoral VA CPB is not possible in the beating heart, even with the use of a multi‐stage venous cannula designed to augment venous drainage. This is because beating hearts continue to receive and pump deoxygenated venous blood as the vena cavae are not clamped. In addition, augmenting pump flows, even for a brief period, trying to improve upper body oxygen saturations can be detrimental because this can significantly increase LV end‐diastolic pressures leading to LV dilatation with subsequent acute pulmonary edema.^22^

In addition, when we use a single multi‐stage femoral venous cannula in a normally functioning beating heart, venous drainage most often will be insufficient. In this situation, oxygenation of the deoxygenated venous blood passing through the heart can be done by placing an additional venous cannula (most often in the right IJV) that delivers oxygenated blood. This describes a venoarterial‐venous (VA‐V) CPB configuration, which necessitates precise regulation of pressures by clamps and flow sensors^27^ and sometimes the use of two separate pumps.^28^ On the other hand, venous drainage is most often adequate in a normally functioning beating heart if two venous cannulae are properly positioned (most often in the femoral vein and IJV).^7^ This describes a venovenous‐arterial (VV‐A) CPB configuration. However, IJV manipulation was not feasible in our fully heparinized patient, with the neck draped and positioned for rigid bronchoscopy. Accurate positioning of two different wires for two venous cannulae requires either fluoroscopy or transesophageal echocardiography (TEE), both of which were not possible in the setting of severe respiratory compromise.

High‐dose beta‐blocker therapy with IV esmolol could have reduced her native LV output. This is useful in her situation by ensuring that the oxygenated blood pumped from the femoral arterial cannula can now reach the aortic arch and aortic root.^22^

Another possible way to ameliorate Harlequin syndrome was to convert to central arterial cannulation of the right axillary artery or ascending aorta, in order for oxygenated blood to be pumped directly into the aortic arch and aortic root.^7^ However, this requires surgical exposure of right axillary artery for direct cannulation or performing a sternotomy, respectively. In our patient, the safest and quickest approach to correct Harlequin syndrome was endotracheal intubation and lung ventilation. At this point, this approach was possible since the tracheal schwannoma was greatly downsized by the ENT surgeon.

VV ECMO support, such as with the femoral vein‐right IJV approach, can prevent Harlequin syndrome from happening. In addition, VV ECMO support requires minimal anticoagulation and has lower incidences of retrograde arterial atheromatous embolization and lower limb ischemia, which are more commonly seen with VA extracorporeal support, whether by VA ECMO or VA CPB. However, VV ECMO is not capable of providing hemodynamic support^29^ and may be complicated by recirculation syndrome, where oxygenated blood delivered to the IVC or SVC may be drained by the other venous cannula if both venous cannulae are misplaced too close to each other or if there is significant TR.^24^ In addition, VV ECMO circuits entail a much higher cost than traditional VA CPB circuits.

Other approaches for VV ECMO support also exist. In femorofemoral VV ECMO support, both femoral veins are percutaneously cannulated. One femoral vein is cannulated using a long single‐stage venous cannula with its tip ending in the RA lumen, acting as a return cannula for oxygenated blood. The other femoral vein is cannulated with a long multi‐stage venous cannula with its tip ending just beneath the RA‐IVC junction. This approach is successfully used in patients requiring VV ECMO support but have no IJV access.^7^ Another approach for VV ECMO support would be to insert a dual‐lumen cannula in the right IJV, under fluoroscopy or TEE guidance.^7^ All these approaches are safe and excellent for oxygenation support in such cases.

Termination of CPB was after tumor biopsy, downsizing, and safe endotracheal tube insertion, as the consensus was that it is safer to continue with CPB support for the whole surgical time period. Although continued CPB support requires full heparinization, fortunately this did not lead to further surgical bleeding. However, whenever possible, all efforts must be made to terminate CPB support after achieving a definitive airway to avoid further surgical bleeding and postoperative coagulopathy.^7^ Normothermia is maintained, if possible, during the whole duration of CPB support in an attempt to terminate CPB quickly and ameliorate postoperative coagulopathy.^7^

Spinal anesthesia can be used for cannulation of the femoral vessels in CPB surgeries.^7^, ^19^ However, this is controversial and uncommonly used by most anesthesiologists who prefer to avoid neuraxial procedures in these cases. Guidelines by the American Society of Regional Anesthesia and Pain Medicine recommend that 1 h at least should pass before full systemic heparinization for CPB after an atraumatic neuraxial procedure has been performed.^30^ Also, it recommends limiting unfractioned heparin doses after performing a neuraxial procedure; therefore, VV ECMO or VA ECMO support are preferred over VA CPB support, as they require only partial heparinization (Table 1).

**TABLE 1:** Summary of the pros and cons of femorofemoral VA CPB, femorofemoral VA ECMO, and VV ECMO support systems. (CPB, cardiopulmonary bypass; VA, venoarterial; VV, venovenous; ECMO, extracorporeal membrane oxygenation).

Feature	VA CPB	VA ECMO	VV ECMO
Cost	+	+++	+++
Heparinization requirements	Full	Partial	Partial
Venous reservoir	Present	Absent	Absent
Possibility of Harlequin syndrome	+	+++	−

## 4 CONCLUSION

Primary tracheal tumors are a rare occurrence. When they present with an obstructive airway they remain a challenge for the anesthesiologist, ENT surgeon, cardiothoracic surgeon, and perfusionist. Careful preoperative surgical planning is necessary and should be conducted swiftly since these patients often present in emergency situations. Despite their potential complications, CPB and ECMO are extremely useful adjuncts and may be safely instituted via peripheral vessels cannulation under ultrasound guidance with local anesthesia and mild intravenous sedation in this select group of challenging patients. Over the last two decades, VV ECMO and VA ECMO are rapidly replacing traditional VA CPB as the preferred modalities for oxygenation support during lung and airway surgeries for which temporary prolonged cessation of mechanical ventilation may be necessary.^7^ However, at this point, both are more expensive than CPB and they require special training for ECMO management.^7^

## AUTHOR CONTRIBUTIONS

**Cynthia Karam:** Conceptualization; formal analysis; investigation; resources; supervision; validation; writing – original draft; writing – review and editing. **Nancy Abou Nafeh:** Conceptualization; formal analysis; investigation; resources; supervision; validation; writing – original draft; writing – review and editing. **Marie T. Aouad:** Conceptualization; formal analysis; investigation; resources; supervision; validation; writing – original draft; writing – review and editing. **Sahar Siddik‐Sayyed:** Conceptualization; formal analysis; investigation; resources; supervision; validation; writing – original draft; writing – review and editing. **Roland Kaddoum:** Conceptualization; formal analysis; investigation; resources; supervision; validation; writing – original draft; writing – review and editing. **Carine Zeeni:** Conceptualization; formal analysis; investigation; resources; supervision; validation; writing – original draft; writing – review and editing. **Sandra Anka:** Conceptualization; funding acquisition; project administration; resources; supervision; validation; writing – original draft; writing – review and editing. **Bashir Shaya:** Conceptualization; formal analysis; investigation; resources; validation; writing – original draft; writing – review and editing. **Amro Khalili:** Conceptualization; formal analysis; investigation; resources; supervision; validation; writing – original draft; writing – review and editing.

## FUNDING INFORMATION

None.

## CONFLICT OF INTEREST STATEMENT

The authors declare that they have no conflict of interests.

## ETHICS STATEMENT

The American University of Beirut Medical Center (AUBMC) Institutional Review Board (IRB) does not require its approval for the involvement of human participants in the publication of case reports.

## CONSENT

Written informed consent was obtained from the patient for the publication of this case report.

## Data Availability

Availability of data and materials: Not applicable.
